# Evidence Mapping Based on Systematic Reviews of Cognitive Behavioral Therapy for Neuropathic Pain

**DOI:** 10.1155/2023/2680620

**Published:** 2023-03-18

**Authors:** Conghui Li, Weiqian Hou, Dongfang Ding, Yujie Yang, Shanshan Gu, Yi Zhu

**Affiliations:** ^1^The Fifth Affiliated Hospital of Zhengzhou University, Zhengzhou, Henan 450000, China; ^2^Academy of Medical Sciences, Zhengzhou University, Zhengzhou, Henan 450000, China; ^3^University of Health and Rehabilitation Sciences, Qingdao, Shandong 266000, China; ^4^Department of Physical Therapy, University of Toronto, Toronto, Ontario, Canada

## Abstract

**Objective:**

This evidence mapping is aimed at identifying, summarizing, and analyzing the available evidence on cognitive behavioral therapy (CBT) for neuropathic pain (NP).

**Methods:**

This study was conducted following the methodology of Global Evidence Mapping (GEM). Searches were conducted in PubMed, Embase, the Cochrane Library, and PsycINFO to identify systematic reviews (SRs) with or without meta-analysis published before February 15, 2022. The authors independently assessed eligibility, extracted data, and evaluated the methodological quality of the included SRs using AMSTAR-2. The results were presented in the tables and a bubble plot based on the identified population-intervention-comparison-outcome (PICO) questions.

**Results:**

A total of 34 SRs met the eligibility criteria. According to the AMSTAR-2, 2 SRs were rated “high,” 2 SRs were rated “moderate,” 6 SRs were rated “low,” and 24 SRs were rated “critically low.” The most common study design utilized to evaluate the efficacy of CBT for NP was the randomized controlled trial. In total, 24 PICOs were identified. Migraine was the most studied population. CBT for NP usually reaches the “potentially better” result at follow-up.

**Conclusions:**

Evidence mapping is a useful way to present existing evidence. Currently, the existing evidence on CBT for NP is limited. Overall, the methodological quality of the included SRs was low. Further improvements in the methodological quality of SRs and more research on the most efficient CBT formats for NP are recommended in the future.

## 1. Introduction

Neurogenic pain (NP) occurs when the somatosensory nervous system is injured or diseased. It can be classified as peripheral or central neuropathic pain depending on where the injury or disease occurs [1, 2]. It is a common chronic pain affecting 6.9% to 10% of the population, and more importantly, it has a significant impact on the quality of life [3]. The mechanisms and physiopathology of neuropathic pain are complex and varied. The potential causes include changes in ion channels, epigenetic regulation, and neuronal function, resulting in the abnormal activity of nerve fibers, peripheral and central sensitization, and so on [4, 5]. Understanding the relationship between physiological mechanisms and clinical manifestations may help improve pain management. At present, the treatment of neuropathic pain includes pharmacological management and nonpharmacological management. Most guidelines for the management of neuropathic pain focus on pharmacotherapy, including anticonvulsants, tricyclic antidepressants, opioids, and drug combinations [6, 7]. Since drugs may lead to adverse reactions and side effects, more and more studies are exploring the effects of nonpharmacological management of neuropathic pain, including psychotherapy, noninvasive neurostimulation therapy, and physiotherapy [8, 9].

Cognitive behavioral therapy (CBT) is a prevailing psychological treatment for chronic pain. CBT was originally developed by Beck to treat people with depression. It is a structured, problem-oriented, and time-limited therapy [10]. As knowledge has evolved, there has been a third wave of cognitive behavioral therapy, which includes various techniques, such as acceptance and commitment therapy (ACT), mindfulness-based cognitive therapy (MBCT), and metacognitive therapy (MCT) [11, 12]. CBT is a class of interventions that modify maladaptive cognitions to change emotional distress and maladaptive behaviors [11, 13, 14]. The effects of CBT in chronic pain may be related to structural changes in gray matter in brain regions associated with pain management and/or in the functional connectivity of these regions [15]. A study of chronic pain found that CBT significantly improved patients' clinical outcomes (treatment outcomes in pain survey, the chronic pain self-efficacy scale, and pain catastrophizing), and that the intrinsic functional connectivity (iFC) between the anterior default mode network and the amygdala/periaqueductal gray was reduced, while iFC between the basal ganglia network and the right secondary somatosensory cortex was increased compared to pain education [16]. In addition, one study found that after CBT intervention, patients had an increase in brain gray matter volume in the dorsolateral prefrontal and sensorimotor cortexes, which was associated with decreased pain catastrophizing [17]. CBT has been used to relieve neuropathic pain associated with spinal cord injury [18, 19], diabetic peripheral neuropathy [20, 21], and HIV-related peripheral neuropathy [22, 23]. These randomized controlled trials have shown that CBT is feasible and has beneficial effects in patients with neuropathic pain. CBT is not a single intervention, and the programs used in these studies included relaxation, breathing techniques, cognitive restructuring, sleep hygiene, behavioral activation, mindfulness, and values clarification [18, 20, 22]. Although there are some differences in specific treatment techniques for different diseases, they all have important similarities. The various techniques are consistent by changing cognitions to reduce maladaptive behaviors [11, 13, 24]. Unfortunately, some studies had small sample sizes or did not have enough confidence in the results. An online cognitive behavioral pain management program has shown to improve worst pain intensity in patients with chemotherapy-induced peripheral neuropathy (CIPN), but the study had a small sample size and a high dropout rate [25]. A study on neuropathic pain after spinal cord injury showed that the pain intensity decreased after CBT, but no intervention effects (time^∗^group interaction) were found, and the power analysis of pain intensity reduction was much lower than expected [19]. A study showed that CBT significantly reduced HIV-related peripheral neuropathic pain, but 57% of the participants did not complete cognitive behavior intervention sessions [23].

Therefore, evidence of the effect of CBT on NP should be further explored and analyzed. Since NP is a clinical description but not a diagnosis and CBT is a class of interventions, systematic reviews (SRs) may not be the most effective in providing the comprehensive summary. This is because SRs are more focused on a specific clinical problem (specific intervention or exposure, or specific disease type). To cover a broad field, evidence mapping has become an emerging tool used to comprehensively identify and summarize current evidence on a topic [26–29]. Therefore, this study used evidence mapping to identify, summarize, and analyze the available evidence on CBT for NP, thereby assisting the clinical decision-making process.

## 2. Materials and Methods

**Figure 1:**
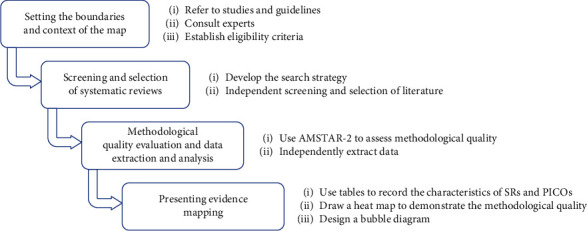
Tasks for performing evidence mapping.

We performed the evidence mapping based on the methodology proposed by the Global Evidence Mapping (GEM) [29] and previous articles [30–32]. The process was divided into four stages (see Figure 1).

### 2.1. Setting the Boundaries and Context of the Map

To construct the evidence map, we reviewed research and guidelines related to CBT and NP, and consulted an expert in pain management. Afterwards, we established the criteria for considering studies according to the PICO: type of study: only SRs (with or without meta-analysis) were included because they provide the most reliable evidence; participants: the samples in the studies should be patients with NP; interventions: the interventions should be CBT; comparisons: the comparisons could be CBT, other treatments, or waiting list; and outcomes: the study results should report pain measured using a variety of clinical validation tools, such as numerical rating scale (NRS), visual analog scale (VAS), and short-form McGill pain questionnaire (SF-MPQ). In addition, SRs focused on physiopathology, prevention, and cost-effectiveness, and non-English studies and other types of research (conference abstracts, books, letters, etc.) were excluded.

### 2.2. Screening and Selection of Systematic Reviews

We searched literature published in PubMed, Embase (Elsevier), the Cochrane Library, and PsycINFO (EBSCO) before February 15, 2022. Mesh terms and free-text terms were used for searching, such as “neuropathic pain”, “neuralgia”, “somatosensory disorders”, and “peripheral nervous system diseases”. In addition, the cited references of included studies were searched to ensure a comprehensive search. The detailed search strategies are available in Supplementary Material 1.

The search results were managed by EndNote X9. After removing duplicates, two reviewers (Li and Ding) screened the titles and abstracts independently and reviewed the full text of relevant literature to make a final decision. Any disagreement between the reviewers was resolved by consensus after consultation and discussion with an independent third reviewer (Zhu).

### 2.3. Methodological Quality Evaluation and Data Extraction and Analysis

The methodological quality of all SRs included in the study was assessed using AMSTAR-2 [33]. AMSTAR-2 contains 16 items, of which items 2, 4, 7, 9, 11, 13, and 15 are the key items. The overall quality of SRs are divided into four levels: “high” means no or one nonkey item missing, “moderate” means more than one nonkey item missing, “low” means one key item with or without nonkey items missing, and “critically low” means more than one key item with or without nonkey items missing.

Then, the following two categories of data were extracted from the SRs:

- General characteristics from SRs: authors, publication year, type of SR (with or without meta-analysis), aim, search date, design and number of included primary studies, and number of included participants
- Characteristics of research questions: the PICO framework was described in detail according to the content of the included SRs. PICO contains four main contents: population (disease diagnosis), intervention (type, intensity, time, and frequency), comparison (type, intensity, time, and frequency), and outcome (impact of intervention)

According to previous studies [30–32], the conclusions reported by authors were divided into five categories: “potentially better” (the conclusions reported a beneficial effect of CBT), “mixed results” (the conclusions of SRs with similar content were inconsistent), “no difference” (there was no significant difference between the intervention and comparison groups), “potentially worse” (the conclusions reported a less beneficial effect in the intervention group), and “unclear” (the conclusions were inconclusive or not reported by the authors)

Two authors (Li and Hou) independently assessed the quality of the methodology and extracted the data. Any disagreement was resolved by consensus after consultation and discussion with an independent third reviewer (Zhu).

### 2.4. Presenting Evidence Mapping

Microsoft Excel 2016 was used to extract the characteristics of the included SRs and identify PICOs. A heat map was used to demonstrate the methodological quality of SRs. In addition, we designed a bubble plot to display multidimensional information. (a) The *x*-axis represented the classification of the author's conclusions (“potentially better,” “mixed,” “no difference,” “potentially worse,” and “unclear”). (b) The *y*-axis represented the evaluation results of AMSTAR-2. (c) The sizes of the bubbles and the numbers on them indicated the number of primary studies contained in the SRs. (d) The colors of the bubbles indicated different PICOs. It should be noted that the same PICO may be derived from multiple primary studies and SRs. If the conclusions of SRs were different, the same PICO would appear at different positions on the *x*-axis. If the methodological quality of the SRs was different, the same PICO would appear at different locations on the *y*-axis.

## 3. Results

**Figure 2:**
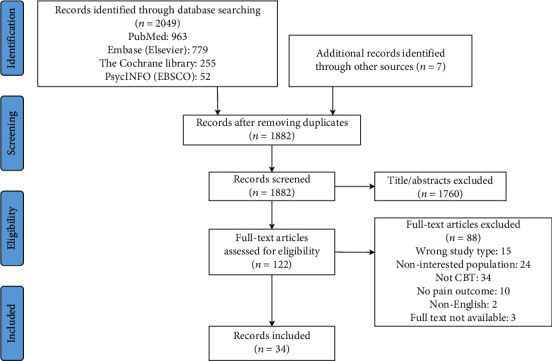
Flow chart for selection process.

The selection of the eligible systematic reviews is shown in Figure 2. The list of the 88 excluded studies and reasons for exclusion are available in Supplementary Material 2.

### 3.1. Characteristics of Systematic Reviews

The characteristics of included SRs are shown in Table 1. We included 34 systematic reviews, 23 of which included a meta-analysis. The earliest review included was published in 2006, and more than half of the reviews were published in the last five years. The sample sizes ranged from 105 to 10586. Three SRs [34–36] did not fully report characteristics, including search date, design of included studies, and participants. According to the evaluation of treatments, 11SRs [37–47] only evaluated CBT-related techniques, 11 SRs [34, 48–57] also evaluated other psychological therapies, 5 SRs [58–62] evaluated nonpharmacological interventions in addition to psychological therapies, 2 SRs [35, 63] evaluated pharmacological and nonpharmacological interventions, and 5 SRs [36, 64–67] evaluated various interventions. Of the available data, 15 SRs [38–40, 43–45, 47, 50, 53–57, 63, 65] included migraine, 7 SRs [34, 35, 52, 60, 64, 66, 67] included burning mouth syndrome, 4 SRs [36, 42, 46, 62] included cancer-related neuropathic pain, 3 SRs [51, 59, 61] included diabetic peripheral neuropathy, 3 SRs [48, 49, 58] included pain after spinal cord injury, and 2 SRs [37, 41] included patients with NP of different causes.

### 3.2. The Methodological Quality of SRs

**Figure 3:**
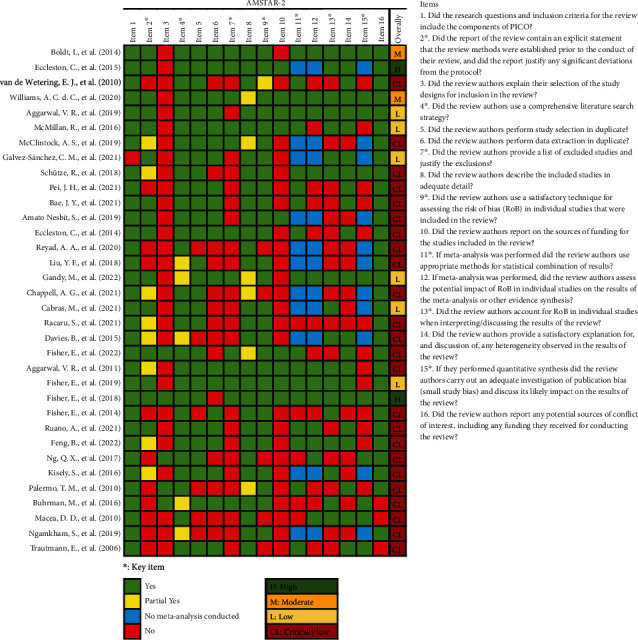
Methodological quality of the included systematic reviews.

According to the AMSTAR-2 scoring criteria (Figure 3), 2 SRs were rated “high.” 2 SRs were rated “moderate,” 6 SRs were rated “low,” and 24 SRs were rated “critically low.” The main reasons for downgrading the SRs were as follows: there was no protocol reported before the review or no explanation for differences from protocol [35–38, 42–47, 51, 52, 55–57, 61, 65–67]; there was no list of excluded studies and reasons for exclusion [34–37, 39, 40, 42, 43, 45–47, 51, 55–57, 59–62, 65–67]; there was no explanation for the study design included in the SRs [34–42, 45–53, 55, 57–62, 64–67]; there was no report of funding sources for studies included in the SRs [35–47, 50, 51, 55–58, 60–62, 65–67]; the risk bias of the included studies was not considered when interpreting or discussing the study results [35–38, 40, 43, 46, 47, 50, 51, 56, 57, 59, 62, 63, 66]; the publication bias was not adequately investigated when quantitative synthesis was made [37, 40, 42, 47, 50–53, 55, 56, 62–64].

### 3.3. Characteristics of PICOs from SRs

After merging the repeated studies extracted from SRs, 24 PICOs were finally extracted. The main characteristics are shown in Table 2. The detailed characteristics of PICOs before merging have been listed in Supplementary Material 3. For the population, 14 PICOs focused on migraines, which were the most extracted PICO questions. For the intervention, 15 PICOs used traditional CBT, 3 PICOs used ACT, 3 PICOs used cognitive restructuring or cognitive therapy, 2 PICOs used MBCT, and 1 PICO used mindfulness. In terms of the delivery type of psychological therapy, 6 PICOs used Internet-based CBT. In addition, PICOs had many forms of comparison, including waiting list/treatment as usual (10 PICOs), placebo/sham control (5 PICOs), education (4 PICOs), pharmacotherapy (2 PICOs), self-administered group (1 PICOs), relaxation (1 PICOs), and standard supportive psychotherapy (1 PICOs).

**Figure 4:**
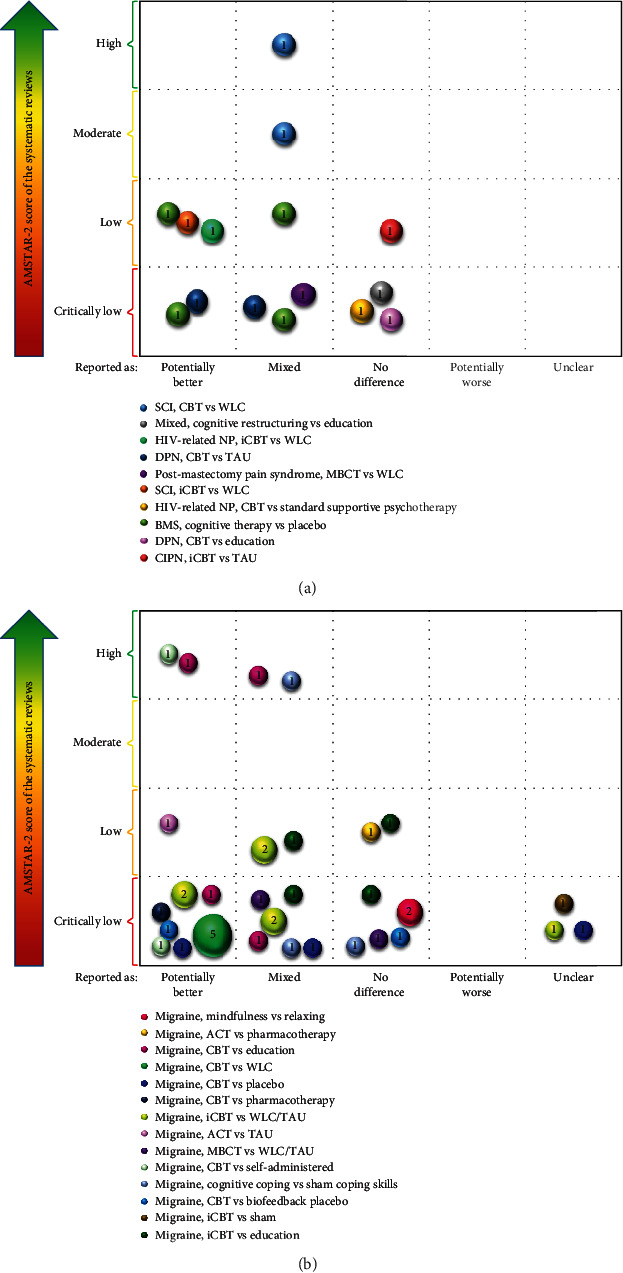
(a) Evidence mapping of the CBT for NP (excluding migraine). (b) Evidence mapping of CBT for migraine. SCI: spinal cord injury; WLC: waiting list control; BMS: burning mouth syndrome; TAU: treatment as usual; iCBT: Internet-based CBT; iACT: Internet-based ACT; DPN: diabetic peripheral neuropathy; CIPN: chemotherapy-induced peripheral neuropathy.

The evidence mapping of the CBT for NP is shown in Figure 4 by visualizing the data in Table 2. The evidence mapping found that the majority of CBT treatments showed “potentially better” or “mixed” results. Among them, CBT seems to have a better effect on patients with neuropathic pain after SCI and burning mouth syndrome (BMS). 12 PICOs included 20 primary studies reported as “potentially better,” two of which involved 2 primary studies that were included in the same high-quality systematic review [54]. 10 PICOs were reported as mixed results due to inconsistent results, demonstrating the limited credibility of the results of these interventions. 10 PICOs included 12 primary studies that showed no difference in CBT compared with the control group. 3 PICOs included 3 primary studies which were rated as “unclear” due to unreported conclusions in the SR. There did not appear to be additional benefit from CBT compared to other types of psychotherapy, such as standard supportive psychotherapy or relaxation. 6 PICOs used Internet-based CBT, but the results of this delivery type were inconsistent. However, it is important to note that most of the studies which had reported the effects of follow-up had concluded that long-term follow-up was potentially beneficial.

## 4. Discussion

There are several ways to synthesize evidence and evaluate the effectiveness of interventions. We used an emerging approach called evidence mapping to visually display information in a user-friendly format [28]. This evidence map reviews studies published up to January 2022 and may be the first evidence map describing CBT for NP. Current research indicates a growing interest in the nonpharmacological treatments for neuropathic pain.

According to the analyzed results from the evidence mapping in this study, only a few studies are focusing alone on CBT for NP. Most SRs included a small number of original studies which focused solely on NP. Therefore, current evidence regarding CBT for NP may be limited. But the studies included were almost always randomized controlled trials, which are the best study design for evaluating the efficacy of treatments [92]. It is important to note that some of these studies were published earlier and had smaller sample sizes. Therefore, large-scale randomized controlled trials are necessary in the future. In addition, no PICO was identified as “potentially worse,” and we cannot rule out the possibility that RCTs with negative conclusions are rarely published [93].

According to our results, the AMSTAR-2 scores of the SRs were generally low. To improve the methodological quality of future SRs, reviewers should pay more attention to the following aspects: to develop and provide an accessible protocol before conducting a review, to provide a list of excluded studies and the reasons for each exclusion, to discuss and explain the impact of risk bias on the results, to analyze and discuss the cause of heterogeneity and its impact on research results, and to report the sources of funding for included studies. Although evidence mapping does not require methodological quality assessment, some research results have suggested that the process is necessary to assess the reliability of conclusions [26, 30].

CBT is not just an effective intervention for people with mental illnesses. According to our analysis, 34.29% of PICOs revealed that CBT is “potentially beneficial” to NP. At the same time, a number of studies reported conclusions with “mixed” (28.57% PICOs) or “no difference” (28.57% PICOs). There were some differences in the conclusions of the current studies, and even the studies with “potentially beneficial” conclusions did not show large effect sizes. It may be due to the difference in CBT techniques, intervention duration, and intervention frequency. However, no study compared the effect of different CBT techniques on NP. Therefore, in the future, while updating the mixed conclusions, reviewers should go further in the efficacy of different techniques, treatment prescriptions, levels of supervision, and many other aspects of CBT for NP to seek better treatment strategies. In addition, we found that CBT was also effective on NP during follow-up, although these studies had a small to moderate effect size [61, 62, 64]. This indicated that patients would take more time to change their cognition and behavior. Differing from pain education, cognitive behavioral therapy is a structured approach. Patients may experience changes in ideas, cognitive processes, pain management behavior, and so on [10, 94]. Therefore, long-term follow-up should be considered when optimizing trial programs in the future.

Telemedicine has become the new way to provide healthcare with technological advancements. We noticed that 5 SRs [41, 44, 45, 50, 53] had evaluated the effectiveness of cognitive behavioral therapy (delivered remotely) for the management of chronic pain. The results of the studies showed that Internet-based CBT produced small but significant improvements. Although there is currently insufficient evidence to draw strong conclusions about the efficacy of Internet-based CBT for NP, these results are promising and can encourage future research in this field. There is no doubt that Internet-based psychological therapies offer many advantages, including addressing distance barriers and reducing health service costs. In the face of the COVID-19 epidemic, healthcare providers are also changing the way of pain management. Therefore, further research is warranted to increase confidence in using Internet-delivered CBT.

## 5. Limitations

This evidence mapping has some limitations. First, although we conducted our literature search in 2022, the included SRs conducted their literature searches in 2021 or earlier. Therefore, we could not investigate studies published recently but have not yet been included in the existing systematic reviews. Nevertheless, we do not believe this limitation would seriously affect the results of evidence mapping. Secondly, we used AMSTAR-2 to assess the methodological quality of the SRs, but not the GRADE to evaluate the evidence level, since our main study objective is finding research gaps to guide further research rather than supporting clinical decision-making. Thirdly, the conclusions of some SRs may be biased due to methodological limitations. However, we provided detailed reports for readers to evaluate each conclusion. Finally, we only included the SRs published in English because of the language barrier.

## 6. Conclusions

There is limited evidence about CBT for NP. Overall, the methodological quality of the included SRs was low. Evidence mapping is a useful method to guide further research to fill in current knowledge gaps. Existing research indicated that remote pain intervention could be effective. In the future, more investigation is needed to explore the optimal CBT formats for NP. Furthermore, the methodological quality of reporting SRs should be improved.

## Figures and Tables

**Table 1:** Characteristics of the included SRs.

Study	Design	Search date	Aim	Design and number of included studies	Participants
Boldt et al. [58]	SRM	March 2011	To appraise the effects of nonpharmacological interventions for chronic pain in spinal cord injury	16 RCT: 16	616
Eccleston et al. [49]	SR	March 2015	To assess the effects of psychological treatments for chronic neuropathic pain in adults	2 RCT: 2	105
van de Wetering et al. [37]	SRM	August 2008	To evaluate the effects of cognitive and behavioral interventions for chronic neuropathic pain	14 RCT: 3 Controlled before-after studies: 3 Uncontrolled before-after studies: 7 Time series analysis: 1	546
Williams et al. [48]	SRM	April 2020	To determine the efficacy and safety of psychological interventions for chronic pain in adults	75 RCT: 75	9401
Aggarwal et al. [34]	SRM	September 2017	To determine the efficacy of self-management interventions for chronic orofacial pain in adults	14 RCT: 14	NA
McMillan et al. [64]	SRM	December 2015	To determine the efficacy and safety of any intervention for burning mouth syndrome	23 RCT: 23	1285 patients included; 1121 patients assessed
McClintock et al. [38]	SR	August 2017	To review the effects of brief mindfulness-based interventions on acute and chronic pain outcomes	20 RCT: 16 Qualitative: 1 Within-group: 3	1740
Galvez-Sánchez et al. [39]	SR	January 2021	To determine the efficacy of acceptance and commitment therapy for central pain sensitization syndromes	21 RCT: 7 Controlled pre-/posttest study: 3 Uncontrolled pre-/posttest study: 2 Randomized controlled trial (pilot study): 2 Uncontrolled trial (pilot study): 1 Pilot study: 2 Open, randomized trial: 1 Randomized pre-/posttest control group design: 1 Longitudinal study: 1 Quasiexperimental, replicated single-case/small group design: 1	1201
Schütze et al. [65]	SRM	November 2016	To investigate treatment-related changes in pain catastrophizing	79 RCT: 79	9914
Pei et al. [47]	SRM	October 2019	To examine the efficacy and safety of mindfulness-based cognitive therapy for chronic pain	8 RCT: 8	433
Bae et al. [40]	SRM	October 2021	To evaluate the efficacy and safety of cognitive behavioral therapy for migraines	11 RCT: 11	621
Amato Nesbit et al. [59]	SR	May 2016	To assess benefits and harms of nonpharmacological interventions for diabetic peripheral neuropathy symptoms	23 RCT: 23	2749
Eccleston et al. [50]	SRM	November 2013	To evaluate the effects Internet-delivered psychological therapies for chronic pain in adults	15 RCT: 15	2012
Reyad et al. [35]	SR	NA	To review pharmacological and nonpharmacological management of burning mouth syndrome	NA	NA
Liu et al. [66]	SR	May 2016	To review treatments for burning mouth syndrome	22 RCT: 22	1131
Gandy et al. [41]	SRM	October 2021	To examine the efficacy of Internet-delivered cognitive and behavioural interventions for chronic pain	36 RCT: 36	5778
Chappell et al. [36]	SR	NA	To review the evidence on postmastectomy pain syndrome	27 RCT: 11 Case series: 8 Prospective randomized comparative study: 3 Double-blinded randomized cross-over study: 3 Prospective open-label single-arm study: 1 Uncontrolled open-label trial: 1	1302
Cabras et al. [60]	SR	August 2020	To evaluate the efficacy of nonpharmacological treatments for burning mouth syndrome	33 RCT: 27 Open clinical trials (OCTs): 6	2055
Racaru et al. [51]	SRM	April 2019	To assess the efficacy of psychological interventions for diabetic peripheral neuropathy	9 RCT: 9	335
Davies et al. [61]	SR	November 2013 and July 2014	To assess the efficacy of physical activity and psychological coping strategies for painful diabetic neuropathy	4 Single blind, RCT: 2 Open label, RCT: 1 Quasiexperimental controlled trial: 1	187
Fisher et al. [63]	SRM	April 2020	To investigate benefits and harms of pharmacological, physical, and psychological interventions for chronic pain in children	122 RCT: 117 Nonrandomized studies: 5	10586
Aggarwal et al. [52]	SRM	October 2010	To assess the efficacy of nonpharmacological psychosocial interventions for chronic orofacial pain	17 RCT: 17	1447
Fisher et al. [53]	SRM	May 2018	To determine the efficacy of psychological therapies delivered remotely for chronic pain in children and adolescents	10 RCT: 10	1023
Fisher et al. [54]	SRM	May 2018	To determine the efficacy of psychological treatments for chronic and recurrent pain in children and adolescents	47 RCT: 47	2884
Fisher et al. [55]	SRM	April 2013	To examine the effects of psychological therapies for management of chronic pain in children	35 RCT: 35	2054
Ruano et al. [62]	SRM	From January 28, 2015, to December 15, 2020	To assess the efficacy of psychological and nonpharmacological treatments for reducing pain in cancer patients	10 RCT: 10	1057
Feng et al. [42]	SRM	December 2020	To examine the efficacy of MBIs on pain in cancer patients	10 RCT: 10	843
Ng et al. [43]	SRM	May 2016	To examine the use of CBT in pediatric migraine	17 RCT: 17	1028
Kisely et al. [67]	SR	January 2016	To review treatments for burning mouth syndrome	24 RCT: 24	1268
Palermo et al. [56]	SRM	August 2008	To quantify the effects of psychological therapies for chronic pain in youth	25 RCT: 25	1247
Buhrman et al. [44]	SRM	March 2015	To evaluate Internet-based treatments based on cognitive behavioural therapy for chronic pain	22 RCT: 22	2354
Macea et al. [45]	SRM	July 2009	To assess Internet-based cognitive behavioral programs for chronic pain	11 RCT: 11	2958
Ngamkham et al. [46]	SR	From 2008 to 2017	To describe the effectiveness of mindfulness interventions for pain and its underlying pathophysiologic mechanisms	6 RCT: 6	760
Trautmann et al. [57]	SRM	July 2004	To describe the up-to-date state of evidence in the psychological treatment of pediatric headaches	23 RCT: 23	935

SR: systematic review; SRM: systematic review with meta-analysis; MBIs: mindfulness-based interventions; CBT: cognitive behavioral therapy; RCT: randomized controlled trial; NA: not available.

**Table 2:** PICOs included in systematic reviews.

PICOs	Population	Intervention	Comparison	Outcomes	Systematic reviews included	Individual studies included in SR			Conclusion
						RCT	Controlled before–after studies	Longitudinal study	
1	SCI	CBT: 10 sessions of 3 hours over 10 weeks	Waiting list	CPG	Boldt et al. [58]^②^, Eccleston et al. [49]^①^, and Williams et al. [48]^②^	Heutink [19]			Mixed
2	SCI	iCBT: 6 sessions, 6 weeks	WLC	BPI, NRS	Gandy et al. [41]^③^	Burke [18]			Potentially better
3	Mixed	Cognitive restructuring session: 8 Time per session: 1.5 h	Pain education	Scale (0–10)	van de Wetering et al. [37]^④^		Ehde and Jensen [68]		No difference
4	HIV-related peripheral neuropathic pain	CBT week: 6 Session: 6 Group time per session: 1 h	Standard supportive psychotherapy	BPI	van de Wetering et al. [37]^④^	Evans et al. [23]			No difference
5	HIV-related peripheral neuropathic pain	iACT: 12 sessions	WLC	BPI	Gandy et al. [41]^③^	Scott [22]			Potentially better
6	BMS	Cognitive therapy: 12-15 sessions, one hour once a week	Attention/placebo return visits 3 times during 12-15 weeks	VAS ranging from 1 to 7	McMillan et al. [64]^③^, Kisely et al. [67]^④^, Liu et al. [66]^④^, Cabras et al. [60]^③^, and Aggarwal et al. [52]^④^	Bergdahl [69]			Potentially better after greater than 3 months
					Liu et al. [66]^④^, Cabras et al. [60]^③^, Aggarwal et al. [52]^④^, Aggarwal et al. [34]^③^, and Reyad et al. [35]^④^	Bergdahl [69]			Mixed
7	Migraine	Mindfulness: 7 min group session (with script), instructed to practice 20 min/day for 2 weeks at home (given handout). Practiced 15 min just before cold pressor task	Relaxing	Pain tolerance and self-reported pain intensity and stress (0–10 scales)	McClintock et al. [38]^④^	Feuille and Pargament [70]			No difference
8	Migraine	ACT: six 90 min small group sessions (once per week for 6 weeks)	TAU	Monthly migraine days	Galvez-Sánchez et al. [39]^③^			Grazzi et al., [71]	Potentially better (significant results at the 6-month follow-up)
9	Migraine	ACT: six 90 min small group sessions (once per week for 6 weeks)	Erenumab: 70 mg (per month as an adjunct to pharmacological prophylaxis)	Monthly migraine days	Galvez-Sánchez et al. [39]^③^			Grazzi et al., [71]	No difference
10	Migraine	MBCT	WLC/TAU	Headache frequency, severity	Pei et al. [47]^④^, Bae et al. [40]^④^	Mansourishad [72]			Mixed
					Bae et al. [40]^④^	Seng [73]			No difference
11	Migraine	CBT: 8 weekly, 1-hour individual sessions, followed by monthly booster sessions of similar duration at weeks 12 and 16 and at the 3-, 6-, and 9-month follow-up points	Education	HA frequency (day/month)	Bae et al. [40]^④^, Ng et al. [43]^④^, Fisher et al. [63]^④^, and Fisher et al. [54]^①^	Powers [74]			Mixed
					Ng et al. [43]^④^, Fisher et al. [54]^①^	Powers [74]			Potentially better at 3 months or later
12	Migraine	CBT	Self-administered group/self-monitoring	Total headache index	Fisher et al. [63]^④^	McGrath [75]			Potentially better
					Fisher et al. [54]^①^	Griffiths [76]			Potentially better
13	Migraine	CBT	Waiting list	Headache diary using 0–5 intensity/headache diary using 0–10 NRS intensity	Ng et al. [43]^④^	Griffiths [76], Kroener-Herwig [77], Larsson [78], Osterhaus [79], and Barry [80]			Potentially better (potentially better at 3 months or later)
14	Migraine	CT (cognitive coping)	Sham coping skills	Headache diary (headache index, frequency, mean duration, and peak intensity)	Fisher et al. [63]^④^	Richter [81]			No difference (follow-up)
					Fisher et al. [63]^④^, Fisher et al. [54]^①^, Fisher et al. [55]^④^, Ng et al. [43]^④^, Palermo et al. [56]^④^, and Trautmann et al. [57]^④^	Richter [81]			Mixed
15	Migraine	CBT	Placebo control	Headache diary using 0–10 NRS intensity	Ng et al. [43]^④^	Passchier [82]			Unclear
				Headache diary using 0–10 NRS intensity	Ng et al. [43]^④^	McGrath [75]			Potentially better at 3 months or later
				Headache diary using 0–5 intensity	Palermo et al. [56]^④^, Trautmann et al. [57]^④^, and Ng et al. [43]^④^	McGrath [75]			Mixed
16	Migraine	CBT	Biofeedback placebo	Headache diary using 0–4 scale (4 being the highest intensity)	Ng et al. [43]^④^	Scharff [83]			Potentially better
				Headache diary using 0–4 scale (4 being the highest intensity)	Ng et al. [43]^④^	Scharff [83]			No difference at 3 months or later
17	Migraine	CBT coping skills	Metoprolol 50–100 mg OM	Headache diary using 0–10 NRS intensity	Ng et al. [43]^④^	Sartory [84]			Potentially better (beneficial at 3 months or later)
18	Migraine	CBTi baseline 2 weeks + 6 weeks (biweekly)	Sham control (lifestyle modification)	HA frequency (days/month), HA severity	Bae et al. [40]^④^	Smitherman [85]			Unclear
19	Migraine	Internet CBT	Waiting list/TAU	Clinical reduction in headache frequency	Fisher et al. [53]^③^, Macea et al. [45]^④^, Palermo et al. [56]^④^, Fisher et al. [55]^④^, and Ng et al. [43]^④^	Connelly [86]			Mixed
				Daily headache record (headache duration, number of hours, and severity on a 4-point scale), PCS, and PGIC	Eccleston et al. [50]^④^	Bromberg [87]			Unclear
				PCS	Schütze et al. [65]^④^, Gandy et al. [41]^③^, and Buhrman et al. [44]^④^	Bromberg [87]			Mixed
				Headache diary using 0–10 NRS intensity	Ng et al. [43]^④^	Hicks [88]			Potentially better (potentially better at 3 months or later)
				Clinical reduction in headache frequency	Fisher et al. [55]^④^, Ng et al. [43]^④^	Connelly [86]			Potentially better at two-month and three-month follow-up
20	Migraine	Online CBT	Education	HA frequency (% of days), HA duration (h/episode), and HA severity (VAS)	Bae et al. [40]^④^, Fisher et al. [53]^③^, and Fisher et al. [63]^④^	Rapoff [89]			Mixed
				HA frequency (% of days), HA duration (h/episode), and HA severity (VAS)	Fisher et al. [53]^③^, Fisher et al. [63]^④^	Rapoff [89]			No difference at 3-month follow-up
21	DPN	CBT one-on-one, 60-minute weekly sessions over 11 weeks	TAU	WHYMPI	Amato Nesbit et al. [59]^④^, Davies et al. [61]^④^, and Racaru et al. [51]^④^	John D Otis [21]			Mixed
				WHYMPI	Davies et al. [61]^④^, Racaru et al. [51]^④^	John D Otis [21]			Potentially better at 4-month follow-up
22	DPN	CBT: 10 weekly sessions of 60 minutes, delivered one-on-one by a doctoral-level psychologist	Education	NRS	Racaru et al. [51]^④^	Kerns [90]			No difference
23	Postmastectomy pain syndrome	MBCT: 8 consecutive weeks, one weekly session of two hours + 45 min exercises at home	WLC	SF-MPQ-2, NRS	Chappell et al. [36]^④^, Ngamkham et al. [46]^④^, Ruano et al. [62]^④^, Feng et al. [42]^④^	Johannsen [91]			Mixed
24	CIPN	iCBT: 10 sessions, 8weeks	TAU	NRS	Gandy et al. [41]^③^	Knoerl [25]			No difference

SCI: spinal cord injury; BMS: burning mouth syndrome; DPN: diabetic peripheral neuropathy; CIPN: chemotherapy-induced peripheral neuropathy; WLC: waiting list control; TAU: treatment as usual; iCBT: Internet CBT; iACT: Internet ACT; CPG: chronic pain grade; BPI: the Brief Pain Inventory; HA: headache; NRS: numerical rating scale; PCS: pain catastrophizing scale; PGIC: Patient Global Impression of Change; VAS: visual analog scale; WHYMPI: West Haven Yale Multidimensional Pain Inventory; SF-MPQ-2: short-form McGill pain questionnaire 2; ^①^high-quality SRs; ^②^moderate-quality SRs; ^③^low-quality SRs; ^④^critically low-quality SR.

## Data Availability

The data that support the findings of this study are from the published literature.

## Supplementary Materials

Supplementary 1 Supplementary Material 1: database search strategies.

Supplementary 2 Supplementary Material 2: articles excluded with reasons.

Supplementary 3 Supplementary Material 3: characteristics of PICOs in the SRs.
