# Supplementary material for: Evidence Mapping Based on Systematic Reviews of Cognitive Behavioral Therapy for Neuropathic Pain
Source: Neural Plast. 2023 Mar 18;2023:2680620. doi: 10.1155/2023/2680620 (PMC10041341; doi:10.1155/2023/2680620)
Supplement: Supplementary 1 — Supplementary Material 1: database search strategies. [file 2680620.f1.docx]

**Database search strategies**

Pubmed:

#1 "Neuralgia"[Mesh] OR "Peripheral Nervous System Diseases"[Mesh] OR "Somatosensory Disorders"[Mesh] OR Neuralgia*[Title/Abstract] OR Neurodynia*[Title/Abstract] OR NP[Title/Abstract]

#2 (central[Title/Abstract] OR complex[Title/Abstract] OR rheumat*[Title/Abstract] OR muscl* OR muscul*[Title/Abstract] OR myofasci*[Title/Abstract] OR nerv*[Title/Abstract] OR neuralg*[Title/Abstract] OR neuropath*[Title/Abstract]) AND (pain*[Title/Abstract] OR discomfort*[Title/Abstract])

#3 (neur*[Title/Abstract] OR nerv*[Title/Abstract]) AND (compress*[Title/Abstract] OR damag*[Title/Abstract])

#4 #1 OR #2 OR #3

#5 "Psychotherapy"[Mesh] OR "Behavior Therapy"[Mesh] OR "Cognitive Behavioral Therapy"[Mesh] OR "Mindfulness"[Mesh] OR "Meditation"[Mesh] OR "Relaxation Therapy"[Mesh] OR "Acceptance and Commitment Therapy"[Mesh] OR "Biofeedback, Psychology"[Mesh] OR "Imagery, Psychotherapy"[Mesh] OR "Hypnosis"[Mesh] OR "Mind-Body Therapies"[Mesh] OR "Psychotherapy, Group"[Mesh] OR Psychotherap*[Title/Abstract] OR Cognitive Behavioral Therap*[Title/Abstract] OR Cognitive Behavior Therap*[Title/Abstract] OR Cognitive behavioural Therap*[Title/Abstract] OR Cognitive Behaviour Therap*[Title/Abstract] OR CBT[Title/Abstract] OR Psychoeducation*[Title/Abstract] OR mindfulness[Title/Abstract] OR meditation[Title/Abstract] OR "acceptance and commitment therapy"[Title/Abstract] OR ACT[Title/Abstract] OR biofeedback[Title/Abstract] OR "Guided imagery"[Title/Abstract] OR Hypnotherap*[Title/Abstract] OR Supportive psychotherap*[Title/Abstract] OR group therap*[Title/Abstract] OR Relaxation Therap*[Title/Abstract]

#6 (Psychotherapeutic[Title/Abstract] OR Cognit*[Title/Abstract] OR behavio*[Title/Abstract] OR mind body[Title/Abstract]) AND (psychotherap*[Title/Abstract] OR therap*[Title/Abstract] OR modif*[Title/Abstract] OR restructur*[Title/Abstract] OR treat*[Title/Abstract] OR intervention*[Title/Abstract] OR technique*[Title/Abstract] OR train*[Title/Abstract] OR counsel*[Title/Abstract] OR strateg*[Title/Abstract] OR education*[Title/Abstract])

#7 #5 OR #6

#8 #4 AND #7

Filters: Meta-Analysis, Systematic Review

Embase:

#1 'neuropathic pain'/exp OR 'peripheral neuropathy'/exp OR 'somatosensory disorder'/exp OR 'neuralgia'/exp

#2 'neurodynia*':ti,ab,kw OR 'np':ti,ab,kw

#3 ((pain* OR discomfort*) NEAR/6 (central OR complex OR rheumat* OR muscl* OR muscul* OR myofasci* OR nerv* OR neuralg* OR neuropath*)):ti,ab,kw

#4 ((neur* OR nerv*) NEAR/4 (compress* OR damag*)):ti,ab,kw

#5 #1 OR #2 OR #3 OR #4

#6 'psychotherapy'/exp OR 'behavior therapy'/exp OR 'cognitive behavioral therapy'/exp OR 'mindfulness'/exp OR 'meditation'/exp OR 'relaxation training'/exp OR 'acceptance and commitment therapy'/exp OR 'mindfulness meditation'/exp OR 'biofeedback'/exp OR 'guided imagery'/exp OR 'hypnosis'/exp OR 'group therapy'/exp

#7 ((cognit* OR behavio* OR psychotherapeutic OR group OR interpersonal OR couple OR relax* OR imaginat* OR hypnotherapeutic OR 'mind body') NEAR/3 (counsel* OR psychotherap* OR rehabilitat* OR therap* OR modif* OR restructur* OR treat* OR intervention* OR technique* OR train* OR strateg* OR education*)):ti,ab,kw

#8 psychotherap*:ti,ab,kw OR cbt:ti,ab,kw OR psychoeducation*:ti,ab,kw OR mindfulness:ti,ab,kw OR meditation:ti,ab,kw OR act:ti,ab,kw OR biofeedback:ti,ab,kw OR 'guided imagery':ti,ab,kw OR hypnotherap*:ti,ab,kw OR 'supportive psychotherap*':ti,ab,kw

#9 #6 OR #7 OR #8

#10 #5 AND #9

Filters: Meta-Analysis, Systematic Review

Cochrane Library:

#1 MeSH descriptor: [Neuralgia] explode all trees

#2 MeSH descriptor: [Peripheral Nervous System Diseases] explode all trees

#3 MeSH descriptor: [Somatosensory Disorders] explode all trees

#4 (neurodynia* OR NP):ti,ab,kw

#5 ((pain* OR discomfort*) NEAR (central OR complex OR rheumat* OR muscl* OR muscul* OR myofasci* OR nerv* OR neuralg* OR neuropath*)):ti,ab,kw

#6 ((neur* OR nerv*) NEAR (compress* OR damag*)):ti,ab,kw

#7 #1 OR #2 OR #3 OR #4 OR #5 OR #6

#8 MeSH descriptor: [Psychotherapy] explode all trees

#9 MeSH descriptor: [Behavior Therapy] explode all trees

#10 MeSH descriptor: [Cognitive Behavioral Therapy] explode all trees

#11 MeSH descriptor: [Mindfulness] explode all trees

#12 MeSH descriptor: [Meditation] explode all trees

#13 MeSH descriptor: [Relaxation Therapy] explode all trees

#14 MeSH descriptor: [Acceptance and Commitment Therapy] explode all trees

#15 MeSH descriptor: [Biofeedback, Psychology] explode all trees

#16 MeSH descriptor: [Imagery, Psychotherapy] explode all trees

#17 MeSH descriptor: [Hypnosis] explode all trees

#18 MeSH descriptor: [Mind-Body Therapies] explode all trees

#19 MeSH descriptor: [Psychotherapy, Group] explode all trees

#20 ((cognit* OR behavio* OR psychotherapeutic OR group OR interpersonal OR couple OR relax* OR imaginat* OR hypnotherapeutic OR mind-body) NEAR/3 (counsel* OR psychotherap* OR rehabilitat* OR therap* OR modif* OR restructur* OR treat* OR intervention* OR technique* OR train* OR strateg* OR education*)):ti,ab,kw

#21 (Psychotherap* OR CBT OR Psychoeducation* OR mindfulness OR meditation OR "acceptance and commitment therapy" OR ACT OR biofeedback OR "Guided imagery" OR Hypnotherap* OR Supportive psychotherap*):ti,ab,kw

#22 #8 OR #9 OR #10 OR #11 OR #12 OR #13 OR #14 OR #15 OR #16 OR #17 OR #18 OR #19 OR #20 OR #21

#23 #7 AND #22

Filter applied: Cochrane Reviews

APA PsycINFO (EBSCO)

S1 (((DE "Neuralgia") OR (DE "Peripheral Neuropathy")) OR (DE "Somatosensory Disorders")) OR (DE "Neuropathic Pain")

S2 TI ( neurodynia* OR NP ) OR AB ( neurodynia* OR NP ) OR KW ( neurodynia* OR NP )

S3 TI ( (pain* OR discomfort*) N6 (central OR complex OR rheumat* OR muscl* OR muscul* OR myofasci* OR nerv* OR neuralg* OR neuropath*) ) OR AB ( (pain* OR discomfort*) N6 (central OR complex OR rheumat* OR muscl* OR muscul* OR myofasci* OR nerv* OR neuralg* OR neuropath*) ) OR KW ( (pain* OR discomfort*) N6 (central OR complex OR rheumat* OR muscl* OR muscul* OR myofasci* OR nerv* OR neuralg* OR neuropath*) )

S4 TI ( (neur* OR nerv*) N4 (compress* OR damag*) ) OR AB ( (neur* OR nerv*) N4 (compress* OR damag*) ) OR KW ( (neur* OR nerv*) N4 (compress* OR damag*) )

S5 S1 OR S2 OR S3 OR S4

S6 ((((((((((DE "Psychotherapy") OR (DE "Behavior Therapy")) OR (DE "Cognitive Behavior Therapy")) OR (DE "Mindfulness")) OR (DE "Meditation")) OR (DE "Relaxation Therapy")) OR (DE "Acceptance and Commitment Therapy")) OR (DE "Biofeedback")) OR (DE "Guided Imagery")) OR (DE "Hypnotherapy") OR (DE "Group Psychotherapy")

S7 TI ( Psychotherap* OR CBT OR Psychoeducation* OR mindfulness OR meditation OR "acceptance and commitment therapy" OR ACT OR biofeedback OR "Guided imagery" OR Hypnotherap* OR Supportive psychotherap* ) OR AB ( Psychotherap* OR CBT OR Psychoeducation* OR mindfulness OR meditation OR "acceptance and commitment therapy" OR ACT OR biofeedback OR "Guided imagery" OR Hypnotherap* OR Supportive psychotherap* ) OR KW ( Psychotherap* OR CBT OR Psychoeducation* OR mindfulness OR meditation OR "acceptance and commitment therapy" OR ACT OR biofeedback OR "Guided imagery" OR Hypnotherap* OR Supportive psychotherap* )

S8 TI ( (cognit* OR behavio* OR psychotherapeutic OR group OR interpersonal OR couple OR relax* OR imaginat* OR hypnotherapeutic OR "mind body") N3 (counsel* OR psychotherap* OR rehabilitat* OR therap* OR modif* OR restructur* OR treat* OR intervention* OR technique* OR train* OR strateg* OR education*) ) OR AB ( (cognit* OR behavio* OR psychotherapeutic OR group OR interpersonal OR couple OR relax* OR imaginat* OR hypnotherapeutic OR "mind body") N3 (counsel* OR psychotherap* OR rehabilitat* OR therap* OR modif* OR restructur* OR treat* OR intervention* OR technique* OR train* OR strateg* OR education*) ) OR KW ( (cognit* OR behavio* OR psychotherapeutic OR group OR interpersonal OR couple OR relax* OR imaginat* OR hypnotherapeutic OR "mind body") N3 (counsel* OR psychotherap* OR rehabilitat* OR therap* OR modif* OR restructur* OR treat* OR intervention* OR technique* OR train* OR strateg* OR education*) )

S9 S6 OR S7 OR S8

S10 S5 AND S9

Filters: Meta-Analysis, Systematic Review
