# Supplementary material for: Evidence Mapping Based on Systematic Reviews of Cognitive Behavioral Therapy for Neuropathic Pain
Source: Neural Plast. 2023 Mar 18;2023:2680620. doi: 10.1155/2023/2680620 (PMC10041341; doi:10.1155/2023/2680620)
Supplement: Supplementary 2 — Supplementary Material 2: articles excluded with reasons. [file 2680620.f2.docx]

**Articles excluded with reasons**

| N | Study | Title | Reason for exclusion |
| --- | --- | --- | --- |
| 1 | Bawa, F. L., et al. (2015) | Does mindfulness improve outcomes in patients with chronic pain? Systematic review and meta-analysis | Non-interested population |
| 2 | Bernier Carney, K. M., et al. (2020) | A Systematic Review of Biological Mechanisms and Chronic Pain Outcomes During Stress Reduction Interventions | Non-interested population |
| 3 | Bostick, G. P. (2017) | Effectiveness of psychological interventions delivered by non-psychologists on low back pain and disability: a qualitative systematic review | Non-interested population |
| 4 | Burgstaller, J. M., et al. (2014) | Treatment efficacy for non-cardiovascular chest pain: A systematic review and meta-analysis | Non-interested population |
| 5 | Cheng, J. O. S. and S. T. Cheng (2019) | Effectiveness of physical and cognitive-behavioural intervention programmes for chronic musculoskeletal pain in adults: A systematic review and meta-analysis of randomised controlled trials | Non-interested population |
| 6 | Chou, R. (2010) | Low back pain (chronic) | Non-interested population |
| 7 | Danon, N., et al. (2021) | Are mind-body therapies effective for relieving cancer-related pain in adults? A systematic review and meta-analysis | Non-interested population |
| 8 | Eccleston, C., et al. (2017) | Interventions for the reduction of prescribed opioid use in chronic non-cancer pain | Non-interested population |
| 9 | Elbers, S., et al. (2022) | Longitudinal outcome evaluations of Interdisciplinary Multimodal Pain Treatment programmes for patients with chronic primary musculoskeletal pain: A systematic review and meta-analysis | Non-interested population |
| 10 | Gross, A., et al. (2012) | Patient education for neck pain | Non-interested population |
| 11 | Hajihasani, A., et al. (2019) | The Influence of Cognitive Behavioral Therapy on Pain, Quality of Life, and Depression in Patients Receiving Physical Therapy for Chronic Low Back Pain: A Systematic Review | Non-interested population |
| 12 | Hall, H. and G. McIntosh (2008) | Low back pain (chronic) | Non-interested population |
| 13 | Makris, U. E., et al. (2014) | Management of persistent pain in the older patient: A clinical review | Non-interested population |
| 14 | Marie, N., et al. (2013) | Optimal patient education for cancer pain: a systematic review and theory-based meta-analysis | Non-interested population |
| 15 | Martinez-Calderon, J., et al. (2020) | Which Interventions Enhance Pain Self-efficacy in People With Chronic Musculoskeletal Pain? A Systematic Review With Meta-analysis of Randomized Controlled Trials, Including Over 12 000 Participants | Non-interested population |
| 16 | May, S. (2010) | Self-management of chronic low back pain and osteoarthritis | Non-interested population |
| 17 | McIntosh, G. and H. Hall (2011) | Low back pain (acute) | Non-interested population |
| 18 | Mistiaen, P., et al. (2016) | The effect of patient-practitioner communication on pain: a systematic review | Non-interested population |
| 19 | Monticone, M., et al. (2015) | Cognitive-behavioral Treatment for Subacute and Chronic Neck Pain: A Cochrane Review | Non-interested population |
| 20 | Monticone, M., et al. (2015) | Cognitive‐behavioural treatment for subacute and chronic neck pain | Non-interested population |
| 21 | Nielson, W. R., et al. (2001) | Biopsychosocial approaches to the treatment of chronic pain | Non-interested population |
| 22 | Petrucci, G., et al. (2021) | Psychological Approaches for the Integrative Care of Chronic Low Back Pain: A Systematic Review and Metanalysis | Non-interested population |
| 23 | Quisel, A., et al. (2005) | Complex regional pain syndrome: Which treatments show promise? | Non-interested population |
| 24 | Romm, M. J., et al. (2021) | A Meta-Analysis of Therapeutic Pain Neuroscience Education, Using Dosage and Treatment Format as Moderator Variables | Non-interested population |
| 25 | Anheyer, D., et al. (2019) | Mindfulness-based stress reduction for treating chronic headache: A systematic review and meta-analysis | Non-interested population |
| 26 | Booth, G., et al. (2022) | What is the content of virtually delivered pain management programmes for people with persistent musculoskeletal pain? A systematic review | Non-interested population |
| 27 | Trindade, I. A., et al. (2021) | Efficacy of Online-Based Acceptance and Commitment Therapy for Chronic Pain: A Systematic Review and Meta-Analysis | Non-interested population |
| 28 | Crawford, C., et al. (2014) | Effectiveness of active self-care complementary and integrative medicine therapies: options for the management of chronic pain symptoms | Not CBT for NP |
| 29 | Merlin, J. S., et al. (2016) | Pharmacologic and non-pharmacologic treatments for chronic pain in individuals with HIV: a systematic review | Not CBT for NP |
| 30 | Morone, N. E. and C. M. Greco (2007) | Mind-body interventions for chronic pain in older adults: a structured review | Not CBT for NP |
| 31 | Amatya, B., et al. (2018) | Non‐pharmacological interventions for chronic pain in multiple sclerosis | Not CBT for NP |
| 32 | Pardos-Gascón, E. M., et al. (2021) | Differential efficacy between cognitive-behavioral therapy and mindfulness-based therapies for chronic pain: Systematic review | Not CBT for NP |
| 33 | Axon, D. R., et al. (2019) | Use of multidomain management strategies by community dwelling adults with chronic pain: evidence from a systematic review | Not CBT |
| 34 | Binder, A. I. (2008) | Neck pain | Not CBT |
| 35 | Brami, C., et al. (2016) | Natural products and complementary therapies for chemotherapy-induced peripheral neuropathy: A systematic review | Not CBT |
| 36 | Brooks, M. R. and B. Golianu (2016) | Perioperative management in children with chronic pain | Not CBT |
| 37 | Carr, D. B., et al. (2004) | Evidence report on the treatment of pain in cancer patients | Not CBT |
| 38 | Cossins, L., et al. (2013) | Treatment of complex regional pain syndrome in adults: a systematic review of randomized controlled trials published from June 2000 to February 2012 | Not CBT |
| 39 | Dalacorte, R. R., et al. (2011) | Pain management in the elderly at the end of life | Not CBT |
| 40 | Delgado, R., et al. (2014) | Assessing the quality, efficacy, and effectiveness of the current evidence base of active self-care complementary and integrative medicine therapies for the management of chronic pain: a rapid evidence assessment of the literature | Not CBT |
| 41 | Duong, S., et al. (2018) | Treatment of complex regional pain syndrome: an updated systematic review and narrative synthesis | Not CBT |
| 42 | Gu, Q., et al. (2018) | Mindfulness Meditation for Primary Headache Pain: A Meta-Analysis | Not CBT |
| 43 | Hou, S., et al. (2018) | Treatment of chemotherapy-induced peripheral neuropathy: Systematic review and recommendations | Not CBT |
| 44 | Lee, C., et al. (2014) | Mind-body therapies for the self-management of chronic pain symptoms | Not CBT |
| 45 | Lee, J. H., et al. (2019) | Nonsurgical treatments for patients with radicular pain from lumbosacral disc herniation | Not CBT |
| 46 | Liampas, A., et al. (2020) | Non-Pharmacological Management of Painful Peripheral Neuropathies: A Systematic Review | Not CBT |
| 47 | Ogle, T., et al. (2020) | Systematic review of the effectiveness of self-initiated interventions to decrease pain and sensory disturbances associated with peripheral neuropathy | Not CBT |
| 48 | Redmer, J., et al. (2013) | Targeting diabetes: The benefits of an integrative approach | Not CBT |
| 49 | Richardson, C. and J. Kulkarni (2017) | A review of the management of phantom limb pain: Challenges and solutions | Not CBT |
| 50 | Rickert, M. M., et al. (2019) | Neuropathic Arthropathy of the Shoulder: A Systematic Review of Classifications and Treatments | Not CBT |
| 51 | Tanay, M. A. L., et al. (2021) | A systematic review of behavioural and exercise interventions for the prevention and management of chemotherapy-induced peripheral neuropathy symptoms | Not CBT |
| 52 | Żyluk, A. and P. Puchalski (2018) | Effectiveness of complex regional pain syndrome treatment: A systematic review | Not CBT |
| 53 | Ammendolia, C., et al. (2022) | Non-operative treatment for lumbar spinal stenosis with neurogenic claudication: an updated systematic review | Multidisciplinary therapy or combination therapy |
| 54 | Baird, E., et al. (2017) | Interventions for treating persistent pain in survivors of torture | Multidisciplinary therapy or combination therapy |
| 55 | Carnes, D., et al. (2012) | Effective delivery styles and content for self-management interventions for chronic musculoskeletal pain: a systematic literature review | Multidisciplinary therapy or combination therapy |
| 56 | Hechler, T., et al. (2015) | Systematic review on intensive interdisciplinary pain treatment of children with chronic pain | Multidisciplinary therapy or combination therapy |
| 57 | Joypaul, S., et al. (2019) | Multi-disciplinary interventions for chronic pain involving education: A systematic review | Multidisciplinary therapy or combination therapy |
| 58 | Kamper, S. J., et al. (2015) | Multidisciplinary biopsychosocial rehabilitation for chronic low back pain: Cochrane systematic review and meta-analysis | Multidisciplinary therapy or combination therapy |
| 59 | Lee, C., et al. (2014) | Multimodal, integrative therapies for the self-management of chronic pain symptoms | Multidisciplinary therapy or combination therapy |
| 60 | Mehta, S., et al. (2013) | Neuropathic pain post spinal cord injury part 1: systematic review of physical and behavioral treatment | Multidisciplinary therapy or combination therapy |
| 61 | Vorobeychik, Y., et al. (2011) | Combination therapy for neuropathic pain: A review of current evidence | Multidisciplinary therapy or combination therapy |
| 62 | Kühne, F., et al. (2016) | [Psychological treatments for pain in cancer patients : A systematic review on the current state of research] | Non-English |
| 63 | Mora Moscoso, R., et al. (2014) | Treatment of central neuropathic pain; Future analgesic therapies: Systematic review | Non-English |
| 64 | Crawford, C., et al. (2014) | The current state of the science for active self-care complementary and integrative medicine therapies in the management of chronic pain symptoms: lessons learned, directions for the future | No pain outcome |
| 65 | Byrnes, K. L. and S. Whillier (2019) | Effects of Nonpharmaceutical Treatments on Symptom Management in Adults With Mild or Moderate Multiple Sclerosis: A Meta-analysis | No pain outcome |
| 66 | Gómara-Toldrà, N., et al. (2014) | Physical therapy after spinal cord injury: A systematic review of treatments focused on participation | No pain outcome |
| 67 | Grossman, P., et al. (2004) | Mindfulness-based stress reduction and health benefits. A meta-analysis | No pain outcome |
| 68 | Pagnini, F., et al. (2014) | Symptom changes in multiple sclerosis following psychological interventions: a systematic review | No pain outcome |
| 69 | Park, R., et al. (2020) | Systematic scoping review of interactions between analgesic drug therapy and mindfulness-based interventions for chronic pain in adults: current evidence and future directions | No pain outcome |
| 70 | van de Graaf, D. L., et al. (2021) | Online Acceptance and Commitment Therapy (ACT) interventions for chronic pain: A systematic literature review | No pain outcome |
| 71 | Acton, C. (2007) | The holistic management of chronic wound pain | Wrong study type |
| 72 | Agoston, A. M. and C. B. Sieberg (2016) | Nonpharmacologic Treatment of Pain | Wrong study type |
| 73 | Chou, R., et al. (2017) | Nonpharmacologic Therapies for Low Back Pain: A Systematic Review for an American College of Physicians Clinical Practice Guideline | Wrong study type |
| 74 | Ducasse, D., et al. (2013) | Burning mouth syndrome: Current clinical, physiopathologic, and therapeutic data | Wrong study type |
| 75 | Gladstone, J. P. and D. W. Dodick (2003) | Current and emerging treatment options for migraine and other primary headache disorders | Wrong study type |
| 76 | Hruschak, V., et al. (2018) | Psychosocial interventions for chronic pain and comorbid prescription opioid use disorders: A narrative review of the literature | Wrong study type |
| 77 | Jones, K. F., et al. (2022) | Pharmacological and Nonpharmacological Management of Chemotherapy-Induced Peripheral Neuropathy: A Scoping Review of Randomized Controlled Trials | Wrong study type |
| 78 | Kerns, J. W., et al. (2006) | Does psychiatric treatment help patients with intractable chronic pain? | Wrong study type |
| 79 | Ketenci, A. and M. Zure (2021) | Pharmacological and non-pharmacological treatment approaches to chronic lumbar back pain | Wrong study type |
| 80 | Lee, C., et al. (2014) | An analysis of the various chronic pain conditions captured in a systematic review of active self-care complementary and integrative medicine therapies for the management of chronic pain symptoms | Wrong study type |
| 81 | Malfliet, A., et al. (2019) | Best evidence rehabilitation for chronic pain part 3: Low back pain | Wrong study type |
| 82 | Moisset, X., et al. (2020) | Pharmacological and non-pharmacological treatments for neuropathic pain: Systematic review and French recommendations | Wrong study type |
| 83 | O'Connell, N. E., et al. (2013) | Interventions for treating pain and disability in adults with complex regional pain syndrome‐ an overview of systematic reviews | Wrong study type |
| 84 | Patton, L. L., et al. (2007) | Management of burning mouth syndrome: systematic review and management recommendations | Wrong study type |
| 85 | Siddall, P. J. and J. D. Loeser (2001) | Pain following spinal cord injury | Wrong study type |
| 86 | Bril, V. (2004) | Filling the gap: Emerging treatments for diabetic neuropathy. | Full text not available |
| 87 | Dodick, D. W. and J. P. Gladstone (2005) | An evidence-based and experience-based approach to acute migraine treatment | Full text not available |
| 88 | Sesel, A. L., et al. (2018) | Efficacy of Psychosocial Interventions for People with Multiple Sclerosis: A Meta-Analysis of Specific Treatment Effects | Data not available |
