# Supplementary material for: Evidence Mapping Based on Systematic Reviews of Cognitive Behavioral Therapy for Neuropathic Pain
Source: Neural Plast. 2023 Mar 18;2023:2680620. doi: 10.1155/2023/2680620 (PMC10041341; doi:10.1155/2023/2680620)
Supplement: Supplementary 3 — Supplementary Material 3: characteristics of PICOs in the SRs. [file 2680620.f3.docx]

**Characteristics of PICOs in the SRs**

| Number | Systematic Reviews | Individual studies included in SR | | | Population | Intervention | Comparison | Outcomes | Effect size | Conclusion  (follow up) |
| --- | --- | --- | --- | --- | --- | --- | --- | --- | --- | --- |
|  |  | RCT | controlled before–after study | Longitudinal study |  |  |  |  |  |  |
| 1 | Boldt, I., et al. (2014) | Heutink 2012 |  |  | SCI | CBT 10 sessions of 3 hours over 10 weeks | waiting list | CPG | MD (short-term):‐2.0 (95% CI ‐9.26 to 5.26);  MD (After three months): 0.40 (95% CI ‐7.3 to 8.1) | unclear |
| 2 | Eccleston, C., et al. (2015) | Heutink 2012 |  |  | SCI | CBT 10 sessions of 3 hours over 10 weeks | waiting list | CPG | MD (post-treatment):‐2.00 (95% CI ‐9.65 to 5.65)  MD (follow-up): 0.40 (95% CI ‐7.69 to 8.49) | no effects |
| 3 | van de Wetering, E. J., et al. (2010) |  | Ehde and Jensen 2004 |  | Mixed | Cognitive restructuring session:8 time per session:1.5h | pain education | scale (0–10) | SMD:‐1.50 (95% CI ‐5.99 to 2.99) | not significant |
|  |  |  | Ehde and Jensen 2004 |  | Mixed | Cognitive restructuring adjusted version session:8 time per session:1.5h | pain education | scale (0–10) | SMD: ‐0.60(95% CI ‐4.13 to 2.93) | not significant |
|  |  | Evans et al. 2003 |  |  | HIV-related peripheral neuropathic pain | CBT Week:6 session:6, Group time per session:1h | standard supportive psychotherapy | BPI | SMD: 0.20(95% CI ‐1.55 to 1.95) | not significant |
| 4 | Williams, A. C. d. C., et al. (2020) | Heutink 2012 |  |  | SCI | CBT | waiting list | Chronic Pain Grade questionnaire pain intensity | SMD: ‐0.14(95% CI ‐0.64 to 0.37) | no effects |
| 5 | Aggarwal, V. R., et al. (2019) | Bergdahl 1995 |  |  | BMS | cognitive therapy 12-15 sessions; one hour once a week | attention placebo | non-validated VAS ranging from 1 to 7 | NA | Not reported |
| 6 | McMillan, R., et al. (2016) | Bergdahl 1995 |  |  | BMS | cognitive therapy 12-15 sessions; one hour once a week | attention/placebo return visits 3 times during 12-15 weeks | VAS ranging from 1 to 7 | MD: ‐3.20(95% CI ‐4.22 to ‐2.18) | a long‐term(> 3 to ≤ 6 months) benefit |
| 7 | McClintock, A. S., et al. (2019) | Feuille and Pargament 2015 |  |  | migraine | Mindfulness of breath 7-min group session (with script), instructed to practice 20 min/day for 2 weeks at home (given handout). Practiced 15 min just before cold-pressor task | Relaxing | pain tolerance and self-reported pain intensity and stress (0–10 scales) | NA | No significant |
|  |  | Feuille and Pargament 2015 |  |  | migraine | spiritualized mindfulness 7-min group session (with script), instructed to practice 20 min/day for 2 weeks at home (given handout). Practiced 15 min just before cold-pressor task | Relaxing | pain tolerance and self-reported pain intensity and stress (0–10 scales) | NA | No significant |
| 8 | Galvez-Sánchez, C. M., et al. (2021) |  |  | Grazzi et al., 2020 | migraine | ACT six 90-min small group sessions (once per week for 6 weeks) | TAU | Monthly migraine days | NA | beneficial(Significant results at the 6-month follow-up) |
|  |  |  |  | Grazzi et al., 2020 | migraine | ACT six 90-min small group sessions (once per week for 6 weeks) | erenumab 70 mg erenumab (per month as an adjunct to pharmacological prophylaxis) | Monthly migraine days | NA | No significant |
| 9 | Schütze, R., et al. (2018) | Bromberg  2012 |  |  | Migraine | Internet CBT  4wk | TAU 4wk | PCS | SMD (Post-Test Outcomes): ‐0.14  SMD (Follow-Up Outcomes): ‐0.38 | No significant |
| 10 | Pei, J. H., et al. (2021) | Mansourishad & Borjali, 2017 |  |  | Migraine | MBCT 8-wk course, once weekly for 2 h sessions Homework: every day | usual care | headache frequency, severity | SMD: ‐0.75(95% CI ‐1.55 to 0.05) | No significant |
| 11 | Bae, J. Y., et al. (2021) | Powers 2013 |  |  | Migraine | CBT 20 weeks | Education | HA frequency (day/month) | MD: ‐4.70(95% CI ‐8.01 to ‐1.39) | beneficial |
|  |  | Seng 2019 |  |  | Migraine | MBCT-M 8–10 weeks (8 sessions) | WL/TAU | HA days/month, Average Attack Intensity /month, Self-reported headache days over a 90 day period, divided by 3, Self-reported average headache attack intensity over a 90 day period (1–10) | MD: 0.60(95% CI ‐1.83 to 3.03) | No significant |
|  |  | Mansourishad 2017 |  |  | Migraine | MBCT 6 weeks | Control | HA Frequency (days/month), HA Intensity, HA Duration (h/month) | MD: ‐6.00(95% CI ‐8.39 to ‐3.61) | beneficial |
|  |  | Smitherman 2016 |  |  | Migraine | CBTi Baseline 2 weeks + 6 weeks (biweekly) | Sham control (Lifestyle modification) | HA Frequency (days/month), HA Severity | NA | Not reported |
|  |  | Rapoff 2014 |  |  | Migraine | CD-Rom Headstrong 4 weeks | Education | HA frequency (% of days), HA duration (hr/episode), HA severity (VAS) | NA | Not reported |
| 12 | Amato Nesbit, S., et al. (2019) | John D Otis 2013 |  |  | diabetic peripheral neuropathy (DPN) | CBT 11 weekly sessions | TAU | WHYMPI | SMD: –0.87 | unclear |
| 13 | Eccleston, C., et al. (2014) | Bromberg 2011 |  |  | migraine | CBT painACTION, Internet based self‐management tool | treatment as usual | Daily Headache Record(headache duration (number of hours) and severity on a 4-point scale), PCS, PGIC | NA | unclear |
| 14 | Reyad, A. A., et al. (2020) | Bergdahl 1995 |  |  | BMS | cognitive therapy 12-15 sessions; one hour once a week | attention/placebo return visits 3 times during 12-15 weeks | VAS | NA | Not reported |
| 15 | Liu, Y. F., et al. (2018) | Bergdahl 1995 |  |  | BMS | cognitive therapy 12-15 sessions; one hour once a week | attention/placebo return visits 3 times during 12-15 weeks | VAS | MD: 3.1(95% CI 2.41 to 3.79) | beneficial |
| 16 | Gandy, M., et al. (2022) | Bromberg  2012 |  |  | Migraine | Internet CBT  4wk | TAU 4wk | PCS | Hedges’ s g=0.384 (95% CI 0.091 to 0.678) | beneficial |
|  |  | Burke 2019 |  |  | SCI | iCBT 6 sessions, 6wk | WLC | BPI, NRS | Hedges’ s g=0.732 (95% CI 0.249 to 1.214) | beneficial |
|  |  | Knoerl 2018 |  |  | CIPN, Chemotherapy-induced peripheral neuropathy | iCBT 10 sessions, 8wk | TAU | NRS | Hedges’ s g=0.494 (95% CI ‐0.077 to 1.065) | No significant |
|  |  | Scott 2021 |  |  | PN in HIV | iACT 12 sessions | WLC | BPI | Hedges’ s g=0.978 (95% CI 0.281 to 1.675) | beneficial |
| 17 | Chappell, A. G., et al. (2021) | Johannsen 2016 |  |  | Post-Mastectomy Pain Syndrome | MBCT 8 weeks | WLC | SF-MPQ-2, NRS | NA | beneficial |
| 18 | Cabras, M., et al. (2021) | Bergdahl 1995 |  |  | BMS | cognitive therapy 12-15 sessions; one hour once a week | attention/placebo return visits 3 times during 12-15 weeks | VAS ranging from 1 to 7 | NA | beneficial |
| 19 | Racaru, S., et al. (2021) | Kerns 2015 |  |  | Diabetic Peripheral Neuropathy | CBT 10 weekly sessions of 60 minutes, delivered one-on-one by a doctoral-level psychologist | education | NRS | SMD (short term): ‐0.23(95% CI ‐0.84 to 0.39) | No significant |
|  |  | Otis 2013 |  |  | Diabetic Peripheral Neuropathy | CBT one-on-one, 60-minute weekly sessions over 11 weeks | usual treatment | BPI, Multidimensional Pain Inventory | SMD (short term): ‐1.01(95% CI ‐2.06 to 0.05)  SMD (medium term): ‐1.54(95% CI ‐2.69 to ‐0.38) | No significant at short term follow-up, beneficial at medium term follow-up |
| 20 | Davies, B., et al. (2015) | Otis 2013 |  |  | Painful diabetic neuropathy | CBT one-on-one, 60-minute weekly sessions over 11 weeks | usual treatment | WHYMPI | NA | beneficial(beneficial at 4 month follow up) |
| 21 | Fisher, E., et al. (2022) | McGrath 1992 |  |  | Migraine | CBT | Self-administered group | total headache index | RR: 2.30(95% CI 1.10 to 4.85) | beneficial |
|  |  | Powers 2013 |  |  | Migraine | CBT 8 weekly, 1-hour individual sessions, followed by monthly booster sessions of similar duration at weeks 12 and 16, and at the 3-, 6-, and9-month follow-up points | Headache education | headache diary | RR: 1.79(95% CI 1.26 to 2.55) | beneficial |
|  |  | Rapoff 2014 |  |  | Migraine | CBT Headstrong lessons 4 weeks, with approximately 1 lesson per day | Education control | Headache frequency, intensity/severity, and duration | RR (post-treatment): 1.10(95% CI 0.46 to 2.62)  RR (follow-up): 1.00(95% CI 0.53 to 1.88) | No significant(No significant at 3-month follow-up) |
|  |  | Richter 1986 |  |  | Migraine | CT(cognitive coping) | Sham coping skills | Headache Diary(Headache Index, Frequency, Mean Duration; and Peak Intensity) | RR (post-treatment): 0.10(95% CI ‐0.66 to 0.86)  RR (follow-up): 0.00(95% CI ‐0.67 to 0.67) | No significant(No significant) |
| 22 | Aggarwal, V. R., et al. (2011) | Bergdahl 1995 |  |  | BMS | cognitive therapy 12 to 15 sessions lasting for 1 hour once a week. | attention placebo | VAS ranging from 1 to 7 | SMD(post‐treatment): - 2.4（95% CI - 3.4 to -1.4）  SMD(follow‐up): - 2.79（95% CI - 3.83 to -1.75） | beneficial(The intensity was further reduced in a 6-month follow-up) |
| 23 | Fisher, E., et al. (2019) | Connelly 2006 |  |  | Migraine | CBT Headstrong CD-ROM 4 weeks, one module per week | waiting list | total pain (headache diary: headache frequency per week, duration and intensity per episode, and headache index composite) | RR: 2.50(95% CI 0.90 to 6.94) | no difference |
|  |  | Rapoff 2014 |  |  | Migraine | CBT Headstrong lessons 4 weeks, with approximately 1 lesson per day | Education control | Headache frequency, intensity/severity, and duration | RR (post-treatment): 1.10(95% CI 0.46 to 2.62)  RR (follow-up): 1.00(95% CI 0.53 to 1.88) | No significant(No significant at 3-month follow-up) |
| 24 | Fisher, E., et al. (2018) | Griffiths 1996 |  |  | Migraine | Cognitive behavioural therapy | Self monitoring | Headache index (averaged intensity) | RR: 3.20(95% CI 1.16 to 8.80) | beneficial |
|  |  | Powers 2013 |  |  | Migraine | CBT 8 weekly, 1-hour individual sessions, followed by monthly booster sessions of similar duration at weeks 12 and 16, and at the 3-, 6-, and 9-month follow-up points | Headache education | headache frequency | RR (post-treatment): 1.79(95% CI 1.26 to 2.55)  RR (follow-up): 1.25(95% CI 1.03 to 1.52) | beneficial(beneficial results at 3-month follow-up) |
|  |  | Richter 1986 |  |  | Migraine | Cognitive coping 1 h of individual therapy weekly | attention placebo | headache diary (pain intensity (rated 0-5 4 times daily), frequency, duration) | NA | Not reported |
| 25 | Fisher, E., et al. (2014) | Connelly 2006 |  |  | Migraine | CBT Headstrong CD-ROM 4 weeks, one module per week | waiting list | headache diary (headache frequency per week, duration and intensity per episode, and headache index composite) | RR (post-treatment): 2.65(95% CI 0.99 to 7.08)  RR (follow-up): 2.94(95% CI 1.12 to 7.70) | no difference(beneficial at two months, three months follow up) |
|  |  | Richter 1986 |  |  | Migraine | Cognitive coping 1 h of individual therapy weekly | attention placebo | headache diary (pain intensity (rated 0-5 4 times daily), frequency, duration) | NA | Not reported |
| 26 | Ruano, A., et al. (2021) | Johannsen 2016 |  |  | breast cancer | MBCT Eight consecutive weeks, one weekly session of two hours + 45 min exercises at home | waiting list | SF-MPQ-2 | SMD(post‐treatment): 0.693（95% CI 0.364 to 1.022）  SMD(follow‐up): 0.774（95% CI 0.409 to 1.139） | beneficial(beneficial at 3-month and 6-month follow-up) |
| 27 | Feng, B., et al. (2022) | Johannsen 2016 |  |  | breast cancer | MBCT Eight consecutive weeks, one weekly session of two hours | waiting list | NRS (intensity, interference) | SMD(post‐treatment): - 0.33（95% CI - 0.70 to 0.04）  SMD(follow‐up): - 0.27（95% CI - 0.64 to 0.10） | no difference(no difference at 3- month, 6-month follow up) |
| 28 | Ng, Q. X., et al. (2017) | Barry 1997 |  |  | Migraine | Group CBT treatment | waiting list | Headache diary using 0–10 numerical rating scale (NRS) intensity | NA | beneficial(beneficial at 3 months or later) |
|  |  | Connelly 2006 |  |  | Migraine | CBT Headstrong CD-ROM 4 weeks, one module per week | waiting list | Headache diary | NA | beneficial(beneficial at 3 months or later) |
|  |  | Griffiths 1996 |  |  | Migraine | Cognitive behavioural therapy | waiting list | Headache diary using 0–5 intensity | NA | beneficial(beneficial at 3 months or later) |
|  |  | Hicks 2006 |  |  | Migraine | Online CBT | medical care wait-list control | Headache diary using 0–10 NRS intensity | NA | beneficial(beneficial at 3 months or later) |
|  |  | Kroener-Herwig 2002 |  |  | Migraine | CBT | waiting list | Headache diary using 0–5 intensity | NA | beneficial(beneficial at 3 months or later) |
|  |  | Larsson 1987 |  |  | Migraine | CBT | waiting list | Headache diary using 0–5 intensity | NA | beneficial(beneficial at 3 months or later) |
|  |  | McGrath 1992 |  |  | Migraine | CBT | Attention placebo control | Headache diary using 0–10 NRS intensity | NA | beneficial(beneficial at 3 months or later) |
|  |  | Osterhaus 1997 |  |  | Migraine | CBT | waiting list | Headache diary using 0–5 intensity | NA | beneficial(beneficial at 3 months or later) |
|  |  | Passchier 1990 |  |  | Migraine | CBT | Placebo control | Headache diary using 0–5 intensity | NA | Not reported |
|  |  | Powers 2013 |  |  | Migraine | CBT 8 weekly, 1-hour individual sessions, followed by monthly booster sessions of similar duration at weeks 12 and 16, and at the 3-, 6-, and 9-month follow-up points | Headache education | Headache diary using 0–10 NRS intensity | NA | no difference (beneficial at 3 months or later) |
|  |  | Richter 1986 |  |  | Migraine | CBT coping skills | Attention placebo control | Headache diary using 0–5 intensity | NA | Not reported |
|  |  | Sartory 1998 |  |  | Migraine | CBT coping skills | Metoprolol 50–100 mg OM | Headache diary using 0–10 NRS intensity | NA | beneficial(beneficial at 3 months or later) |
|  |  | Scharff 2002 |  |  | Migraine | CBT | Biofeedback placebo | Headache diary using 0–4 scale (4 being the highest intensity) | NA | beneficial(no difference at 3 months or later) |
| 29 | Kisely, S., et al. (2016) | Bergdahl 1995 |  |  | BMS | cognitive therapy 12-15 sessions; one hour once a week | attention/placebo return visits 3 times during 12-15 weeks | VAS | NA | beneficial at two and 12 months follow-up |
| 30 | Palermo, T. M., et al. (2010) | Connelly 2006 |  |  | Migraine | CBT Headstrong CD-ROM 4 weeks, one module per week | waiting list | Headache diary | NA | beneficial |
|  |  | McGrath 1992 |  |  | Migraine | CBT 8 weekly sessions (60 minutes each in clinic group) | Attention placebo control | Headache diary using 0–5 intensity | NA | beneficial |
|  |  | Richter 1986 |  |  | Migraine | Cognitive coping 6 sessions, 60 minutes | Attention placebo control | Headache diary | NA | Not reported |
| 31 | Buhrman, M., et al. (2016) | Bromberg  2012 |  |  | Migraine | CBT  4wk | TAU 4wk | Daily headache record, PCS | SMD: - 1.212（95% CI – 1.556 to – 0.867） | beneficial |
| 32 | Macea, D. D., et al. (2010) | Connelly 2006 |  |  | Migraine | CBT Headstrong CD-ROM 4 weeks (6 sessions) | Standard medical care waiting-list | Shorter headaches duration, improvement at headache Index, lower headaches frequency and headache intensity | SMD: 0.65（95% CI – 0.02 to 1.31） | no difference |
| 33 | Ngamkham, S., et al. (2019) | Johannsen 2016 |  |  | breast cancer | MBCT Eight consecutive weeks, one weekly session of two hours | waiting list | MPQ-SF | NA | beneficial |
| 34 | Trautmann, E., et al. (2006) | Richter 1986 |  |  | Migraine | Cognitive coping 6 sessions | placebo treatment 6 sessions | Headache diary(pain intensity (rated 0-5 4 times daily), frequency, duration) | Hedges’ s g = 0.66（95% CI 0.28 to 1.04） | beneficial |
|  |  | McGrath 1992 |  |  | Migraine | CBT 8 sessions | Attention placebo control | Headache diary using 0–5 intensity | NA | Not reported |
|  |  |  |  |  |  |  |  |  |  |  |

SCI: spinal cord injury; BMS: burning mouth syndrome; DPN: diabetic peripheral neuropathy; CIPN: chemotherapy-induced peripheral neuropathy; WLC: waiting list control; TAU: treatment as usual; iCBT: Internet CBT; iACT: Internet ACT; CPG: chronic pain grade; BPI: the Brief Pain Inventory; HA, headache; NRS: numerical rating scale; PCS: pain catastrophizing scale; PGIC: Patient Global Impression of Change; VAS: visual analog scale; WHYMPI: West Haven Yale Multidimensional Pain Inventory; SF-MPQ-2: short form McGill pain questionnaire 2; MD: mean difference; SMD: standardized mean difference; RR: Risk Ratio; NA: not available
